# In Vitro Antifungal Activity and Mechanism of Ag_3_PW_12_O_40_ Composites against *Candida* Species

**DOI:** 10.3390/molecules25246012

**Published:** 2020-12-18

**Authors:** Xinming Zhang, Tianzhan Zhang, Shuanli Guo, Yang Zhang, Rongtian Sheng, Ruimeng Sun, Lixia Chen, Ruijuan Lv, Yanfei Qi

**Affiliations:** 1 School of Public Health, Jilin University, Changchun 130021, China; xmzhang19@mails.jlu.edu.cn (X.Z.); guoshuanli@autobio.com.cn (S.G.); yangzhang19@mails.jlu.edu.cn (Y.Z.); shengrt19@mails.jlu.edu.cn (R.S.); Sunrm20@mails.jlu.edu.cn (R.S.); chenlx20@mails.jlu.edu.cn (L.C.); Lvrj20@mails.jlu.edu.cn (R.L.); 2 College of Material Science and Engineering, Jilin Jianzhu University, Changchun 130021, China; Zhang080809@yeah.net

**Keywords:** polyoxometalates, pomposites, antifungal activity, ergosterol, RT-PCR

## Abstract

Fungal infections pose a serious threat to human health. Polyoxometalates (POMs) are metal–oxygen clusters with potential application in the control of microbial infections. Herein, the Ag_3_PW_12_O_40_ composites have been synthesized and verified by Fourier transform infrared (FT-IR) spectrum, transmission electron microscopy (TEM), scanning electron microscope (SEM), elemental analysis, and X-ray diffraction (XRD). The antifungal activities of Ag_3_PW_12_O_40_ were screened in 19 *Candida* species strains through the determination of minimum inhibitory concentration (MIC) by the microdilution checkerboard technique. The minimum inhibitory concentration (MIC_50_) values of Ag_3_PW_12_O_40_ are 2~32 μg/mL to the *Candida* species. The MIC_80_ value of Ag_3_PW_12_O_40_ to resistant clinical isolates *C. albicans* HL963 is 8 μg/mL, which is lower than the positive control, fluconazole (FLC). The mechanism against *C. albicans* HL963 results show that Ag_3_PW_12_O_40_ can decrease the ergosterol content. The expressions of ERG1, ERG7, and ERG11, which impact on the synthesis of ergosterol, are all prominently upregulated by Ag_3_PW_12_O_40_. It indicates that Ag_3_PW_12_O_40_ is a candidate in the development of new antifungal agents.

## 1. Introduction

Fungal infections pose a serious threat to human life and health, especially among immunocompromised patients, such as organ transplant patients, implanted medical devices, and those with indwelling catheters [1,2,3]. The *Candida* species such as *C. albicans*, *C. glabrata*, *C. parapsilosis*, *C. tropicalis,* and *C. krusei* are the main pathogens that may cause severe mucosal and systemic infections [2]. Effective antifungal drugs are the key to the treatment of *Candida* infections [4]. Azoles, such as fluconazole, itraconazole, posaconazole, and voriconazole are considered as the first-line treatment of patients with *Candida* infections [5,6]. However, the broad utilization of azoles has led to drug resistance, which is an increasing problem in clinic isolates [7,8]. Therefore, it is important to develop novel antifungal agents.

Composite antimicrobial materials are a new class of functional materials developed on the basis of composites, which usually consist of antimicrobial metals and inorganic or organic carriers. Antimicrobial metals are the key compositions in composite antimicrobial materials, and their ability varies greatly. Some studies in the literature report that inorganic nanoparticles, such as silver and ZnO, can be used as antifungal agents [9,10,11,12]. At present, silver antimicrobial materials are hot spot researched and widely used in medical catheters, bone adhesives, and other medical devices [13]. Polyoxometalates (POMs) are early transition metal oxygen anion clusters. They have received wide attention because of their versatile structures and various applications in chemistry, materials science, redox, and medicine [14,15,16,17,18]. So far, various biological effects of POMs have been reported, such as penetrating cell walls, inducing cell-apoptosis, inhibition of virus binding to a receptor, and inhibition of the bacterial growth [19]. For example, Yamase et al. found that the polyoxotungstates with Keggin, lacunary Keggin, Wells–Dawson, double-Keggin, and Keggin–sandwich structures enhanced the antibacterial activity of β-lactam antibiotics on drug-resistant *Staphylococcus aureus* [17,19,20,21]. With regard to the antifungal activity of POMs, we and other groups found that polyoxotungstates has antifungal activity against various *Candida* species and agricultural fungal pathogens [22,23]. Thus, we hypothesize that the combination of silver and polyoxotungstates may product new composites with antifungal properties.

In a recent report, we described the antibacterial activity of Ag_3_PW_12_O_40_ against *Staphylococcus aureus* and biofilm [23]. It is interesting to study the antimicrobial spectrum of Ag_3_PW_12_O_40_. In this work, we demonstrate that Ag_3_PW_12_O_40_ composites have antifungal activities against several strains of drugs-susceptible and resistant *Candida* species. We further show that the Ag_3_PW_12_O_40_ composites can influence the ergosterol contents and related genes expressions of the FLC-resistant *Candida* strain.

## 2. Results

### 2.1. Characterization of Ag_3_PW_12_O_40_ Composites

**Figure 1:**
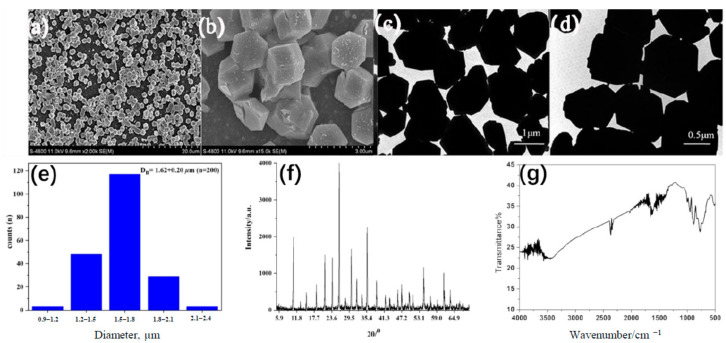
The characterization of Ag_3_PW_12_O_40_ composites. (**a**,**b**) SEM photomicrographs (**c**,**d**) TEM photomicrographs (**e**) The diameter, (**f**) XRD pattern, and (**g**) FT-IR spectrum of the Ag_3_PW_12_O_40_ composites.

The Ag_3_PW_12_O_40_ composites were identified by FT-IR spectrum, TEM, XRD, and SEM. TEM and SEM images indicated the Ag_3_PW_12_O_40_ composites form aggregates of a symmetric dodecahedral shape (Figure 1a–d), having an average diameter about 1.6 μm (Figure 1e). The surface zeta potential was measured as −29.0 ± 2.3 mV. The formation of Ag_3_PW_12_O_40_ composites are confirmed from the X-ray diffraction pattern (Figure 1f). The crystal structure of Ag_3_PW_12_O_40_ composites are the same as the reported H_3_PW_12_O_40_ powder pattern (JCPDS Card File75-2125) [24]. It could be said that silver salts retained the lattice framework present in the parent acid, and there was no significant change in the crystal structure for the Ag_3_PW_12_O_40_ salt preparations process.

The FT-IR spectrum of Ag_3_PW_12_O_40_ has the characteristic asymmetric stretching vibration peaks at 1002, 952, 883, 771, 586, and 516 cm^−1^, which were attributed to *ν*(W-O_terminal_) and *ν*(W-O_bridge_-W) of polyoxoanion, as shown in Figure 1g. The characteristic peaks of Ag_3_PW_12_O_40_ were nearly consistent with the reported H_3_PW_12_O_40_ date in the literature. The other strong features at 3448 cm^−1^ were assigned to the water molecules.

### 2.2. Antifungal Susceptibility Testing

The Ag_3_PW_12_O_40_ composites were assessed in suspension against the standard and clinical isolated strains of *Candida albicans*, *Candida glabrata*, *Candida krusei*, *Candida parapsilosis*, and *Cryptococcus tropicalis* with fluconazole (FLC) as positive control, as shown in Table 1. The MIC values of Ag_3_PW_12_O_40_ were various on different fungal strains. Different strains have different sensitivities to Ag_3_PW_12_O_40_ and FLC, and for some strains, especially for some clinical resistant strains, Ag_3_PW_12_O_40_ had higher activity than that of FLC. The MIC_50_ and MIC_80_ values of Ag_3_PW_12_O_40_ were 2–32 μg/mL and 4–128 μg/mL, respectively. On account of the FLC-resistant clinic separation, *C. albicans* HL 963 was susceptible to Ag_3_PW_12_O_40_; further research studies were focused on the antifungal activities and the mechanism of Ag_3_PW_12_O_40_ against *C. albicans* HL 963.

### 2.3. Inhibitory Effect of Ag_3_PW_12_O_40_ Composites on C. albicans HL 963 

**Figure 2:**
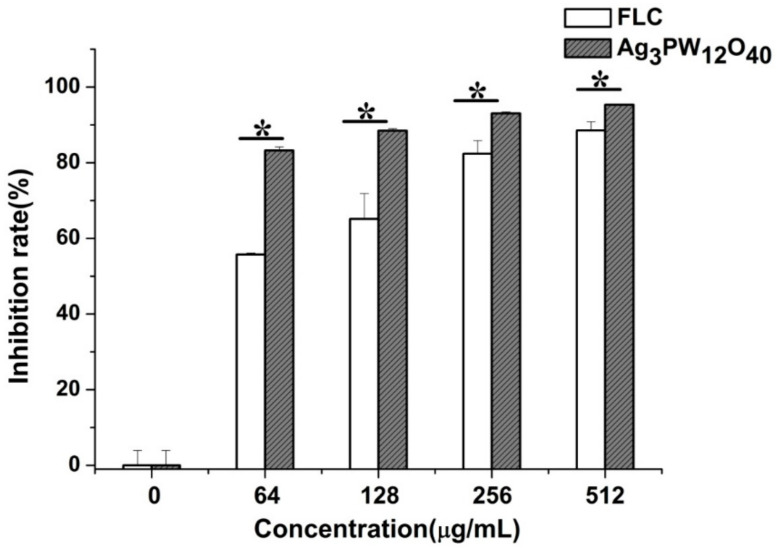
Inhibitory effect of Ag_3_PW_12_O_40_ and FLC on *C. albicans* HL963 in different doses by MTS assay. The experiment was performed in triplicate. Data were represented as mean ± SD. * *p* < 0.05 for Ag_3_PW_12_O_40_ vs. FLC control.

The effect of Ag_3_PW_12_O_40_ and FLC against *C. albicans* HL 963 in different concentrations was further proved by the MTS ((3-(4,5-dimethylthiazol-2-yl)-5-(3-carboxymethoxyphenyl)-2- (4-sulfophenyl)-2H-tetrazolium)) method. As shown in Figure 2, after treatment by Ag_3_PW_12_O_40_, the viability of *C. albicans* HL 963 cells have a significant reduction than in the negative control group (*p* < 0.05). The inhibition ratio of *C. albicans* HL 963 treated by Ag_3_PW_12_O_40_ at 48 h reached the peak values 83.21 ± 0.94%, 88.4 7± 0.51%, 93.01 ± 0.42%, and 95.32 ± 0.11% with the concentrations of 64, 128, 256, and 512 μg/mL, respectively. The inhibitory rates of the same dose of FLC against *C. albicans* HL 963 were 55.74 ± 0.38%, 65.13 ± 6.69%, 82.34 ± 3.47%, and 88.54 ± 2.28%, respectively, proving that the in vitro antifungal activity of Ag_3_PW_12_O_40_ was better than that of FLC on *C. albicans* HL 963.

### 2.4. Growth Inhibition Curves

**Figure 3:**
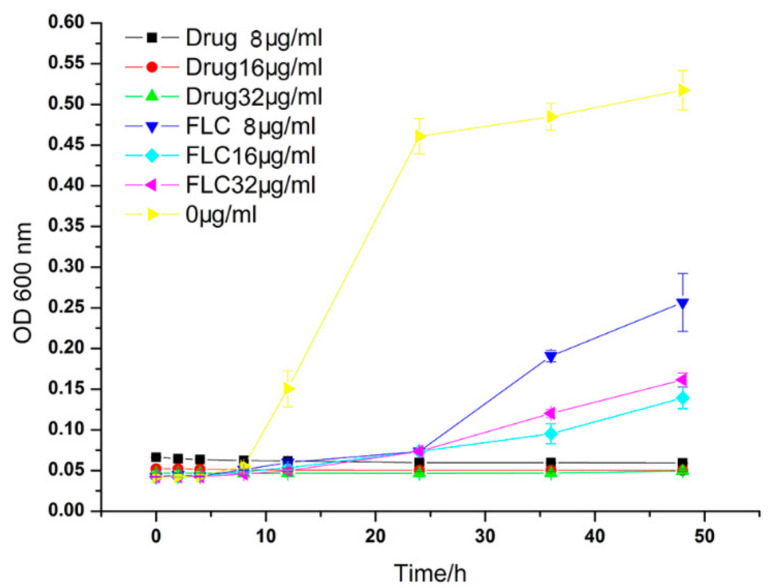
Inhibitory effect of Ag_3_PW_12_O_40_ on *C. albicans* HL963 in different time and different doses. Data are presented as the mean ± SD of three independent experiments.

The growth inhibition effect on the *C. albicans* HL 963 with various concentrations of the Ag_3_PW_12_O_40_, negative control (culture medium without durgs), and FLC at different times is shown in Figure 3. The number of fungi began to proliferate rapidly from the 10th h in the control group; in contrast, the fungi treated with Ag_3_PW_12_O_40_ and FLC was significantly delayed and began to grow 24 h later. After treatment for 24 h, the inhibit delay curve quickly increased up in FLC groups, especially in the low-dose group (8 μg/mL). Meanwhile, the growth of fungi treated with the same dose of Ag_3_PW_12_O_40_ was significantly inhibited, and no reduction of inhibition was observed with the extension of time. All three concentrations of Ag_3_PW_12_O_40_ could inhibit the proliferation of *C. albicans* HL 963.

### 2.5. LIVE/DEAD Assays

**Figure 4:**
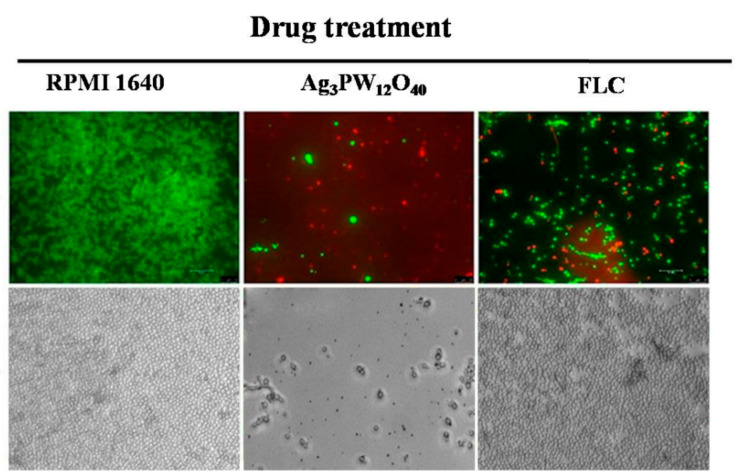
Fluorescent microscopy and optical microscopy of HL963 cells treated with 64 μg/mL of Ag_3_PW_12_O_40_ and equivalent dose of FLC for 24 h, the fluorescent microscopy images were stained with acridine orange (AO) (red) and ethidium bromide (EB) (green). Scale bar: 30 μm.

The cells were stained by acridine orange (AO) and ethidium bromide (EB) double-fluorescent staining. AO can penetrate the intact cell membrane, embed the nuclear DNA, and make it emit bright green fluorescence. However, EB can only penetrate the damaged cell membrane, embed nuclear DNA, and fluoresce orange-red. After culturing 24 h, the fungus was treated with 64 μg/mL Ag_3_PW_12_O_40_ and FLC for 24 h. AO and EB were used to double stain *C. albicans*, and the cell morphology was observed under a fluorescence microscope. The results are shown in Figure 4; after incubation for 24 h, the fungal grew well in the RPMI 1640 group, while cell death (dead cells were stained red) occurred in the Ag_3_PW_12_O_40_ and FLC groups. The massive dead cells were found in the Ag_3_PW_12_O_40_ group, meaning that Ag_3_PW_12_O_40_ is more effective at killing fungi. The result is consistent with the result of the MTS method. In the bright-field images, we observed that *C. albicans* HL 963 formed a very dense cell layer and grew well in the untreated group. Meanwhile, in Ag_3_PW_12_O_40_ and FLC groups at the same dose, the FLC group still had a dense cell layer and compact arrangement. However, in the Ag_3_PW_12_O_40_ group, the number of cells was significantly reduced, and the cells became dispersed. The results showed that Ag_3_PW_12_O_40_ has better antifungal properties than FLC.

### 2.6. Assessment of Ergosterol Content

**Figure 5:**
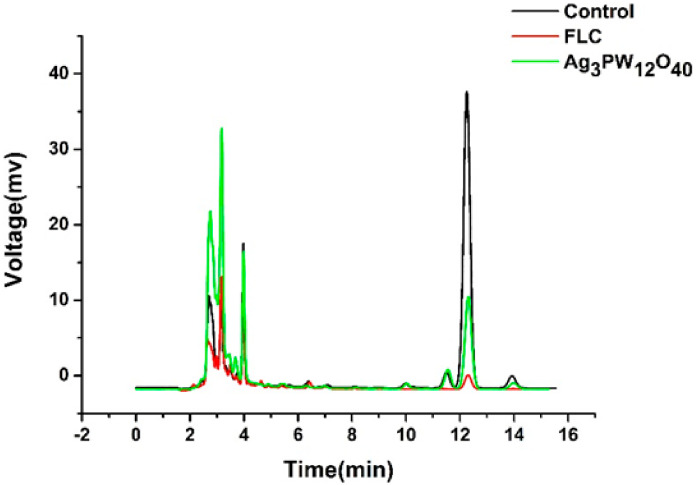
HPLC graphs of ergosterol in *C. albicans* HL963 treated by RPMI1640, FLC, and Ag_3_PW_12_O_40_. The ergosterol extractions of negative control, FLC, and the Ag_3_PW_12_O_40_ group were dissolved with methanol and diluted into 10, 10, and 1 mL, respectively. Each graph displayed three repeated experiments.

**Figure 6:**
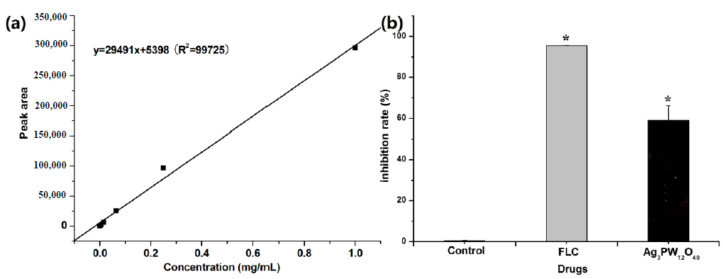
Concentration changes of ergosterol in *C. albicans* HL963 treated with 32 μg/mL of Ag_3_PW_12_O_40_ and 8 μg/mL of FLC after 24 h using the HPLC method. (**a**) The liner relationship between the concentration of ergosterol and the peak area measured by HPLC. (**b**) Inhibition rate of ergosterol after different treatments. Data are presented as the mean ± SD of three independent experiments. * *p* < 0.05 for the Ag_3_PW_12_O_40_ or FLC vs. control.

The HPLC results showed that the retention time of ergosterol was about 12.60 min (Figure 5). Ergosterol contents were extracted from control, FLC, and the Ag_3_PW_12_O_40_ composites after 24 h treatment. The ergosterol contents were 2.20 ± 0.15, 0.10 ± 0.10, and 0.79 ± 0.12 mg/mL, respectively (*p* < 0.05) (Table 2).The standard curve was linear (R^2^ = 0.9945). As shown in Figure 6, the Ag_3_PW_12_O_40_ and FLC treatment for *C.albicans* HL 963 resulted in the ergosterol content reduction of 59.08 ± 7.23% and 95.43 ± 0.19% (*p* < 0.05). The results showed that one of the inhibitory effects of Ag_3_PW_12_O_40_ was through the reduction of ergosterol content on the membrane of *C. albicans*.

### 2.7. The Level of Ergosterol Biosynthesis-Related Genes

**Figure 7:**
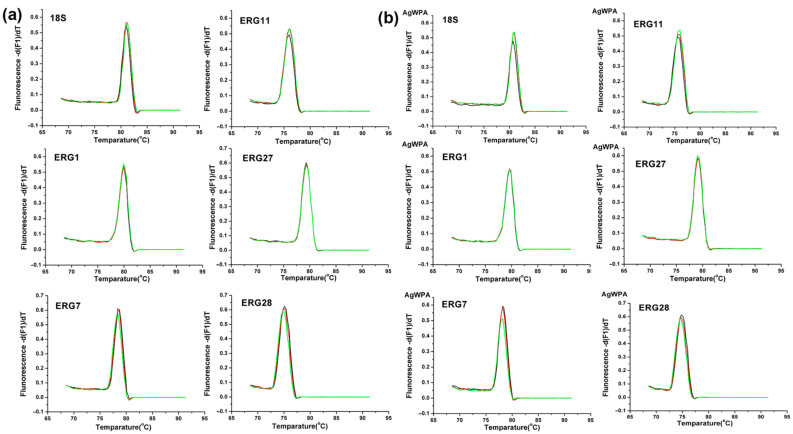
Melt curves of reference gene and target genes in *C. albicans* HL963 treated with (**a**) RPMI1640 or (**b**) Ag_3_PW_12_O_40_.

**Figure 8:**
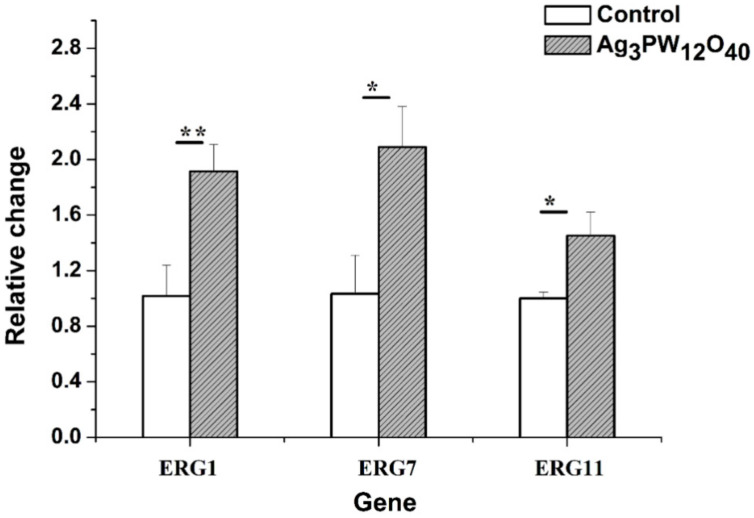
Expression of ERG genes is increased in clinical isolates *C. albicans* HL963. RT-PCR was performed using RNA extracted from cells grown for 24 h treated with 8 μg/mL Ag_3_PW_12_O_40_ composites. All data are normalized to an internal control and are expressed as fold induction relative to the expression level in strain. (* *p* < 0.05 for the Ag_3_PW_12_O_40_ vs. RPMI1640 control ** *p* < 0.01 for the Ag_3_PW_12_O_40_ vs. RPMI1640 control).

To further investigate the mechanism of Ag_3_PW_12_O_40_ reduction of ergosterol biosynthesis, real-time PCR was used to evaluate the expression of five important genes involved in ergosterol biosynthesis. The *C. albicans* HL 963 cells were treated with 8 μg/mL of Ag_3_PW_12_O_40_ for 24 h; then, total RNA was extracted, and cDNA was synthesized by reverse transcription. This cDNA was used as a template for a series of real-time PCRs. The melt curves of reference gene and target genes in *C. albicans* HL 963 treated with RPMI1640 or Ag_3_PW_12_O_40_ are shown in Figure 7. As shown in Figure 8, the expression of ERG1, ERG7, and ERG11 was significantly upregulated with the fold change relative to control of 1.91 ± 0.19, 2.09 ± 0.29, and 1.45 ± 0.17, respectively. 

## 3. Discussion

Invasive fungal diseases have been increasing in recent years. *Candida* spp. is the main pathogen causing infection, ranging from superficial mucous membranes to the whole body. Although several potent antifungal agents are available to treat *Candida* spp., the drug-resistant newstrains frequently emerge. 

Polyoxometalates (POMs) are a kind of metal–oxygen clusters, which are formed by the self-assembly of a transition metal and an oxygen atom and form an inorganic compound having a spatial network structure with many different biological activities than mononuclear complexes. As a new candidate drug, these compounds have the advantages of simple synthesis, low cost, flexible design and modification of molecules, low side effects, and excellent physical and chemical properties (surface charge distribution, molecular shape, pH, solubility, etc.). There are few research studies on the antimicrobial activity of POMs. Yamase group conducted relevant studies and found that some polyoxometalates have good antibacterial activity. For example, decavadate can selectively inhibit *Streptococcus pneumoniae*; Keggin, Keggin deficiency, Double Keggin and Keggin sandwich polytungstate showed a strong synergistic effect with β-lactam antibiotics and synergistic resistance against methicillin-resistant *Staphylococcus aureus* (MRSA), which can improve the antibacterial activity of β-lactam antibiotics against MRSA [20,25,26]. The antifungal effects of polyoxometalates are less studied. In 2002, Sun et al. reported that a series of polymetallic tungstate had antibacterial activity against *Fusarium graminae*, which mainly caused field gramineous crop diseases [27]. In 2011, Chen et al. reported the antimicrobial activity of polyoxometalates on agricultural food fungi [28]. Our group is mainly focused on the design and synthesis of polyoxometalates and their biological activities research in interdisciplinary fields. According to our reports previously, more than 10 polyoxometalates were screened for antifungal susceptibility on 44 strains of human pathogenic fungi, and five POMs were found to have potential antifungal activities [22]. In this study, the antifungal activity of Ag_3_PW_12_O_40_ composites has been evaluated. The results showed that Ag_3_PW_12_O_40_ showed high activity against fungal strains. In particular, it showed an inhibitory effect on FLC-resistant fungus, such as HL 963 (Table 1). An MTS method was further used to evaluate the antifungal activity of Ag_3_PW_12_O_40_ against HL 963. The result is similar to that from the broth microdilution method. The antifungal activity of Ag_3_PW_12_O_40_ is dose-dependent. In comparison with the growth inhibition curves (Figure 3), the Ag_3_PW_12_O_40_ groups had significantly higher inhibition rates than those in the FLC groups (*p* < 0.05). Meanwhile, the AO and EB double fluorescent staining proved that the Ag_3_PW_12_O_40_ group had more dead cells than that in the FLC group (Figure 4).

Moreover, the structure of the Ag_3_PW_12_O_40_ seems to be important for the antifungal activity. POMs have various types of structures, such as Keggin, Wells–Dawson, Anderson, Lindqvist, sandwich Keggin, Preyssler, Strandberg, and Krebs et al. The Keggin–structural polyoxotungstates are known to exhibit various biological activities such as the anti-human immunodeficiency virus, respiratory syncytial virus (RSV), herpes simplex virus (HSV-1, HSV-2), and human cytomegalovirus (HCMV) [29,30,31]. Herein, the PW_12_O_40_^3−^ anions in Ag_3_PW_12_O_40_ also owns a Keggin structure, which may deduce the high antifungal activities. 

In addition, our previous work show that the cytotoxicity of Ag_3_PW_12_O_40_ against L-02 (human normal liver) cells was low (the 50% inhibition concentration value of 274.8 µg/mL). The cytotoxicity value is 8.6–137.4 times higher than the MIC_50_ values (2–32 μg/mL) on the fungi cells in this work [23]. 

Ergosterol is an important component throughout the fungal cell membranes, which distinguishes fungi from bacteria, plant, and animal cells. It plays a vital role in many biological functions such as maintaining cell integrity, regulating membrane fluidity and the cell cycle. Thus, the ergosterol biosynthesis pathway is a significant target of most existing antifungal agents; for instance, fluconazole, itraconazole, amphotericin B, terbinafine, etc. [32]. In this work, the effect of Ag_3_PW_12_O_40_ on the ergosterol content was investigated. Similar to fluconazole, Ag_3_PW_12_O_40_ had also an inhibitory effect on the ergosterol content (Figure 6). Five of the essential genes involved in ergosterol biosynthesis were conducted by real-time PCR. Genes ERG1 (1.91 ± 0.19), ERG7 (2.09 ± 0.29), and ERG11 (1.45 ± 0.17) were unregulated (Figure 8) by Ag_3_PW_12_O_40_. The content of ergosterol decreased with the upregulation of three genes. These results are consistent with previous reports in which *C. albicans* was treated with azole [33,34]. A compensatory response to re-establish the plasma membrane ergosterol levels may account for the observed upregulation of the ERG genes in the ergosterol pathway [2]. When sterol levels are reduced, the expression of ergosterol biosynthesis (ERG) genes are substantially increased. So, the results shown that Ag_3_PW_12_O_40_ may interfere with the expression of ergosterol biosynthesis related genes, decreasing ergosterol content and destroying *Candida albicans* cells. 

In conclusion, Ag_3_PW_12_O_40_ exhibited a potent antifungal effect through mechanisms associated with membrane disruption. Therefore, Ag_3_PW_12_O_40_ is a potential candidate for the development of new antimicrobial agents.

## 4. Materials and Methods

### 4.1. Materials

All the chemicals were analytical grade reagents and used without further purification. The RPMI-1640 with l-glutamine, without sodium bicarbonate (Sigma, Mendota Heights, MN, USA), buffered to pH 7.0 with 165 mM morpholine propanesulfonic acid (MOPS) (Sigma) was used for MIC determination and liquid culture of fungal strains. FLC was obtained from The TCI Company. Ergosterol standard was obtained from Dr. Ehrenstorfer Company (Augsburg, Germany). Prime script RT reagent kit (TaKaRa, Shiga, Japan) was used for reverse transcription. SYBR Green I (Roche, Basel, Switzerland) was used for real-time PCR reactions. 

### 4.2. Synthesis of Ag_3_PW_12_O_40_ Composites

Ag_3_PW_12_O_40_ was synthesized and purified by modifying the published methods [35]. The synthesis process was as follow: 3 mL aqueous solution of AgNO_3_(0.33 M) was added dropwise at a rate of 1 mL/min to 3.35 mL of H_3_PW_12_ O_40_ ethanol–water mixture (0.1 M), under vigorous stirring for 4 h in the dark at room temperature. A white colloidal solution was formed, and the Ag_3_PW_12_O_40_ could be obtained by the evaporation of this solution at 80 °C. Then, the white product of Ag_3_PW_12_O_40_ was treated in a muffle furnace at 300 °C for 2 h. Elemental analysis (%) calcd for Ag_3_PW_12_O_40_: Ag, 10.11; P, 9.57; W, 68.92(%); found: Ag, 10.07; P, 9.61; W, 68.86 (%). IR (KBr pellet, cm^−1^): 3459, 3157, 2708, 2639, 1619, 1087, 996, 954, 892, 829, 765, 731, 513.

### 4.3. Physical Characterization of Ag_3_PW_12_O_40_ Composites

The IR spectrum in the range 400–4000 cm^−1^ was recorded on an Alpha Centaurt FT/IR Spectrophotometer using KBr pellets (Shimadzu, Japan). Ag, P, and W were determined by an inductively coupled plasma optical emission spectrometer (ICP-OES, Optima 7000 DV, Perkin Elmer). The morphology and size of the Ag_3_PW_12_O_40_ composites were acquired by transmission electron microscopy (TEM, JEM-1011, Tokyo, Japan) with a working voltage at 100 kV and a scanning electron microscope (SEM, HITACHI S4800, Tokyo, Japan) was used to characterize the morphology of the samples. Powdered materials were deposited on adhesive tape fixed to specimen tabs and ion-sputter-coated with gold using a BAL-TEC SCD 005 Sputter Coater prior to SEM measurements. The analyses were done under an acceleration voltage of 30 kV, a beam current of 20 nA and a spot size of 1 μm. The X-ray powder diffraction method was carried out in a D/max-α power diffractometer (Rigaku, Tokyo, Japan) using Cu-Kα monochromatic radiation (*λ* = 1.5418 Å). Confocal laser scanning microscope (CLSM) images were obtained from an Olympus FV1000 equipped with 488, 559 nm one-photon laser. Particles’ surface charge (ζ-potential, mV) and diameter were characterized using a Zetasizer Nano Z dynamic light scattering detector (Malvern Instruments Ltd., Worcestershire, UK). Prior to the measurements, composites were diluted to 1 mg/mL with deionized water.

### 4.4. Isolation and Culture Conditions of Fungi

The strains used in this study included 15 *Candida albicans* (HL 973, HL 963, HL 996, HL 27, HL 3929, HL 3973, HL 3863, HL 3084, HL 3961, HL 17034, HL 3916, HL 3974, HL 3970, HL 3968, and ATCC 90028), 1 *Candida glabrata* (HL 981), 1 *Candida krusei* (HL 981), 1 *Candida parapsilosis* (ATCC 22019), and 1 *Cryptococcus tropicalis* (ATCC 750). The strains HL were isolated from patients with clinical fungal infection in Changchun QianWei hospital (China). The *Candida* species were preliminarily identified according to the colored colony morphology on CHRO Magar *Candida* medium (CHRO Magar Co., Paris, France) which was used to confirm the *Candida* species. The reference strains, *Candida tropicalis* ATCC 750, *C. parapsilosis* ATCC 22,019, and *C.albicans* ATCC 90,028 were purchased from American Type Culture Collection. All isolates were incubated at 35 °C and subcultured onto Sabouraud dextrose agar (SDA, Conda) at 4 °C in School of Public Health, Jilin University, China.

### 4.5. Determination of MIC of Ag_3_PW_12_O_40_ Composites

ATCC 22,019 is the quality control strain, which was used in each susceptibility test to ensure quality control. The minimal inhibitory concentration (MIC) values of Ag_3_PW_12_O_40_ and FLC were determined for all the *Candida* strains using the broth microdilution protocol of the Clinical and Laboratory Standards Institute (CLSI) methods (M38-A) [36]. Briefly, the fungal strains were added to sabouraud dextrose broth (SDB) and cultured with shaking at 35 °C and 180 rpm for 18 h and after diluted to 0.4–5 × 10^4^ cells/mL by RPMI 1640. The 96-well plates were prepared by dispensing into each well 100 μL of RPMI-1640 broth. A 100 μL of drugs initially prepared at the concentration of 512 μg/mL was added into each of the first wells, followed by 2-fold serial dilution to obtain a concentration range of 0.25~256 μg/mL. To this was separately added 100 μL of 0.4~5 × 10^4^ cells/mL fungal cell suspensions. The 11th well containing 100 μL medium without drugs and fungal cell was used as the empty control. The last well containing 100 μL fungal cell suspensions without drugs was used as the negative control. The final volume in each well was 200 μL. After 15 s of oscillation, the plates were incubated at 35 °C for 48 h. The absorbance was measured at 600 nm by using a microplate reader (Biotek Co., Winooski, VT, USA). MIC_80_ or MIC_50_ were determined as the lowest concentration of the drugs that inhibited growth by 80% or 50% compared with drug free wells. The inhibitory rate = (1 − OD_drug_ − OD_empty control_/OD_negative control_ − OD_empty control_) × 100%.

### 4.6. MTS-Reduction Assay

The antifungal activity of Ag_3_PW_12_O_40_ on the *C. albicans* HL 963 determined by the MTS assay as described in the literature. Briefly, *C. albicans* HL 963 plated on 96-well plates at a density of 1.0 × 10^6^ cells/mL. After 24 h, the dilutions of Ag_3_PW_12_O_40_ and FLC at different doses (64, 128, 256, and 512 µg/mL) were added and incubated for 48 h. The *C. albicans* HL 963 cells in the negative control group were treated with the same volume of medium. To evaluated cell viability, an MTS assay was performed according to the manufacturer’s instructions (Promega, Madison, WI, USA). The cells were incubated in the dark for another 25 min at 37 °C. Then, using a multichannel pipette, we removed 80 µL of the resulting colored supernatant from each well and transfered it into the corresponding wells of a new microtiter plate. Microplate absorbance was measured at a wavelength of 490 nm on a microplate reader (Biotek Co., Winooski, VT, USA).

### 4.7. Growth Inhibition Curves

The *Candida albicans* strain was cultured in SDB liquid medium at 35 °C overnight. *C.albican* HL 963 was diluted at the starting inoculum of 1 × 10^6^ cells/mL in glass tubes. Different concentrations of the Ag_3_PW_12_O_40_ (8, 16, 32 μg/mL) and FLC (8, 16, 32 μg/mL) were added into tubes. At scheduled time points (0, 2, 4, 8, 12, 24, 36 and 48 h) after incubation in an orbital shaker (about 180 rpm) at 35 °C, a 100 μL aliquot was removed from every solution and at 600 nm with a microtiter plate reader (BioTek, Winooski, VT, USA), and background optical densities were subtracted from that of each well.

### 4.8. LIVE/DEAD Assay

Fungal suspension was prepared from *Candida albicans* after 2 times of activation and diluted to 1–5 × 10^6^ CFU/mL. The sterilized coverslip was put into 6-well plate, and 2 mL fungal solution was added to each well. After incubation for 24 h, the cells were washed 3 times with PBS. The Ag_3_PW_12_O_40_ and FLC with a final concentration of 64 g/mL were added as the experimental group, and the RPMI 1640 was added as the control group. The prepared 6-well plates were incubated at 35 °C for 24 h, and the coverslip was cleaned with PBS 3 times. AO (Acridine Orange) (5 μL, 100 mg/L) and EB (ethidium bromide) (5 μL, 100 mg/L) were mixed with lightless condition; then, we added it to the coverslip, straining in darkness for 30 s. A fluorescence inverted microscope was used to observe and photograph. AO and EB were prepared before the experiment.

### 4.9. Assessment of Ergosterol Content

*C. albicans* HL 963 were treated with Ag_3_PW_12_O_40_ at 35 °C for 24 h. The cells were centrifuged and washed with PBS. A 0.5 g wet weight of cell mixed with PBS and fresh saponifier was saponified at 80 °C for 1 h and extracted by petroleum ether. Then, the extract was volatilized to dryness at 60 °C. The dry residues were dissolved by 0.5 mL methanol and filtered through 0.45 μm micro membrane. Then, we determined the quantification of ergosterol in samples with or without the drugs by comparing peak areas of samples to a standard curve generated from HPLC-grade ergosterol. A standard curve of HPLC-grade ergosterol (Dr. Ehrenstorfer Co., Augsburg, Germany) consists of 0.001, 0.004, 0.015, 0.0625, 0.25, and 1 mg/mL. Ergosterol contents were analyzed using LC-20AB prominence Liquid Chromatography (Shimadzu Co., Kyoto, Japan) including a Shimadzu C_18_ column (250 mm × 4.6 mm, 5 μm). Eluent was methanol/water (97/3, 100% HPLC grade). The flow rate was 1 mL/min. Temperature was 35 °C. SPD-20AV prominence UV/VIS Detector (Shimadzu, Kyoto, Japan) was used to detect UV at 282 nm [37]. The ergosterol inhibition ratio = (1 − ergosterol content of treated cells/ergosterol content of untreated cells) × 100%.

### 4.10. Real-Time PCR

Real-time PCR was used to measure the transcriptional expressions of the genes involved in ergosterol biosynthesis of *C. albicans* treated with Ag_3_PW_12_O_40_. Total RNA was extracted using the hot phenol method as previously described [38]. Reverse transcription was conducted in a total volume of 20 μL with Primescript RT reagent kit (TaKaRa, Shiga, China). Real-time PCR reactions were performed with SYBR Green I (Roche, city, China), using qTOWER 2.0 PCR system (Analytic Jena AG, Jena, Germany). The primer sequences used in real-time PCR were listed in Table 3, using 18S rRNA as the internal control. The expression level of each gene in the Ag_3_PW_12_O_40_-treated sample relative to that of the untreated sample was calculated using the 2^−^^△△Ct^ method.

### 4.11. Statistical Analysis

Data were expressed as mean ± SD (standard deviation) and analyzed by SPSS 16.0 statistical software (IBMCorp, Armonk, NY, USA) (significance was established at *p* < 0.05). All experiments were performed in triplicate. Statistical significance was evaluated by the one-way analysis of variance (ANOVA) and Student’s *t*-test. LSD post hoc analyses of multiple comparisons were statistically analyzed.

## 5. Conclusions

In summary, the Ag_3_PW_12_O_40_ composite is a potential candidate for the development of a new broad spectrum antifungal agent. Future research may focus on trying to explore the antimicrobial mechanism of Ag_3_PW_12_O_40_ and the synthesis of new POM-based compounds.

## Figures and Tables

**Table 1:** Minimum inhibitory concentration (MIC) values (μg/mL) of fluconazole (FLC) and Ag_3_PW_12_O_40_ against fungi.

Strains	MIC_80_		MIC_50_	
	FLC	Ag_3_PW_12_O_40_	FLC	Ag_3_PW_12_O_40_
*C.albicans*				
HL 973	64	128	16	16
HL 963	64	8	4	2–4
HL 996	2	8	1	4
HL 27	2	8	1	4–8
HL3929	>256	64	>256	32
HL3973	16	32	8	16
HL3863	16	64	8	16
HL3084	16	64	4	4
HL3961	4	8	2	4
HL17034	8	64	4	32
HL3916	128	8	8	4–8
HL3974	16	4	0.5	2–4
HL3970	16	64	0.5	8
HL3968	32	32	4	8
ATCC 90028	1	16	0.25	8–16
*C. glabrata*				
HL 981	>256	8	128–256	4
*C. krusei*				
HL 946	>256	8	>256	2
*C.parapsilosis*				
ATCC 22019	2	16	1	4
*C. tropicalis*				
ATCC 750	4	4–8	4	2

**Table 2:** Ergosterol content of *C. albicans* HL 963 treated with or without drugs. Data are presented as the mean ± SD of three independent experiments. * *p* < 0.05 for FLC and Ag_3_PW_12_O_40_ vs. control.

	Concentration (μg/L)	Ergosterol Content (mg/mL)
Control	-	2.20 ± 0.153
FLC	8	0.10 ± 0.003 *
Ag_3_PW_12_O_40_	32	0.79 ± 0.118 *

**Table 3:** Primers used for real time PCR.

Gene	Primer Sequence (5′-3′)	Size (bp)
18S	F: TCTTTCTTGATTTTGTGGGTGG	150
	R: TCGATAGTCCCTCTAAGAAGTG	
ERG1	F: AAGGGCAAAGGTCATGTGTT	121
	R: CGTTAGCAGCAGAAGGAGGT	
ERG7	F: TTATGCGTCGATGTTTGCAT	117
	R: CCACCGTCTGGAAGTTGTTT	
ERG11	F: TTTGACCGTTCATTTGCTCA	110
	R: GCAGCATCACGTCTCCAATA	
ERG27	F: TTGCTGCTGCTTTAGGTCAA	110
	R: GTCCAGACCAGTGCTGTCAA	
ERG28	F: GCAAGAACTTTTGGAACTTGG	117
	R: TGCAGCAATAGCAAATGTGA	
